# Robot-Assisted Removal of a Recurrent Left Ventricular Myxoma

**DOI:** 10.1016/j.atssr.2022.10.009

**Published:** 2022-10-21

**Authors:** Yuji Kawano, Douglas A. Murphy, Michael E. Halkos

**Affiliations:** 1 Department of Cardiac Surgery, MedStar Washington Hospital Center, Washington, DC; 2 Department of Cardiac Surgery, Emory St Joseph’s Hospital, Atlanta, Georgia

## Abstract

Ventricular myxomas are rare compared with atrial myxomas. Unlike with atrial myxomas, it is usually challenging to visualize a ventricular mass because of the complex structures in the left ventricle. We report the case of a 44-year-old woman who had a recurrent left ventricular myxoma 8 years after the initial surgical removal. We successfully performed reoperative tumor resection with a robot-assisted approach through the mitral valve. The robot-assisted approach was extremely effective for visualization of the tumor and precise instrumentation without injuring the surrounding structures.

Cardiac myxomas are rare, benign primary tumors of the heart that can cause a variety of symptoms, depending on size, location, and mobility. Most are found in the left atrium, and those in the left ventricle are extremely rare. Whereas surgical resection of the tumor is the “gold standard” treatment, it is generally challenging to visualize the tumor because of its location surrounded by complex structures in the left ventricle. Commonly, this operation is performed by median sternotomy through the mitral valve, the aortic valve, or sometimes the direct ventricular incision. However, concerns for potential damage to the valvular structure or left ventricular (LV) function remain, especially when the exposure of the tumor is not sufficient for tumor removal. We report the case of a 44-year-old woman who had a recurrent LV myxoma that was successfully removed by a robot-assisted approach through the mitral valve.

The patient is a 44-year-old woman with a history of ovarian cancer, left renal angiomyolipoma, and multiple episodes of transient ischemic attack as well as stroke due to LV myxoma followed by surgical resection through right thoracotomy 8 years ago. She presented to our institution after surveillance echocardiography showed a recurrent LV mass. On the transesophageal echocardiogram, the tumor was measured as 14 × 17 mm, located in the left ventricle along the inferior posterior wall (Figure 1).

**Figure 1:**
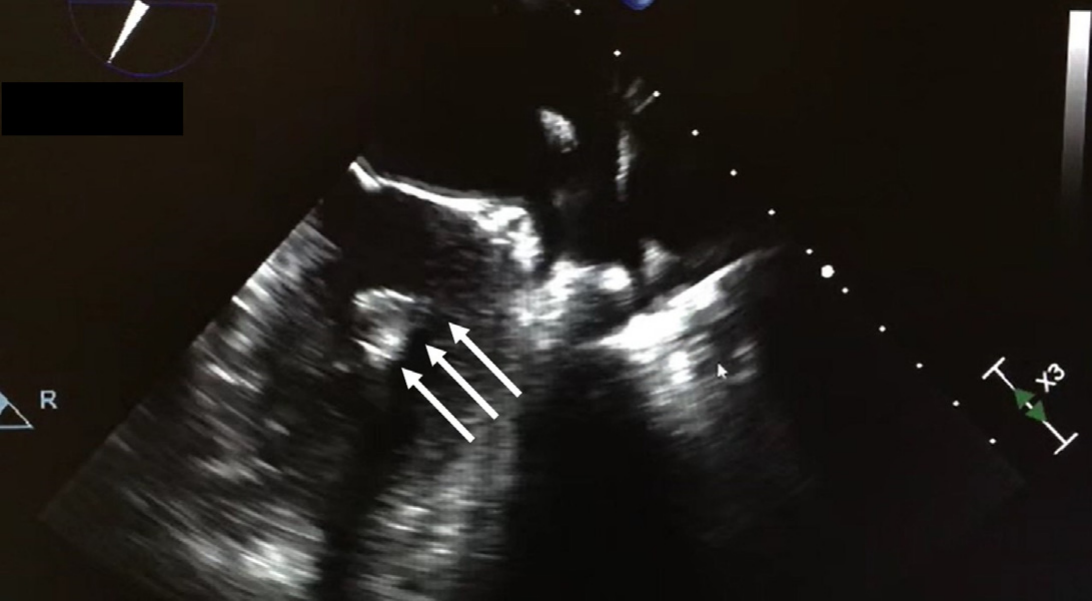
Preoperative transesophageal echocardiogram showing the left ventricular mass on the inferolateral wall (arrows).

Given her previous stroke and the recurrence of LV tumor, surgical resection was indicated. We decided to perform operation by using our previously described lateral endoscopic approach with robotics (LEAR) technique.^1^

After informed consent was obtained, the patient was taken to the operating room and placed in supine position. Following the induction of anesthesia, she was prepared and draped in a sterile fashion. A 2-cm right thoracotomy incision was made superior to the previous right thoracotomy incision. For the robotic port placement, all sites of port placement were relatively free of any significant adhesions. A left femoral cutdown was performed, and the left femoral artery and vein were exposed. A 17F Bio-Medicus (Medtronic) venous cannula was placed in the right internal jugular vein. Then, a 25F femoral venous cannula was placed in the left common femoral vein. A 23F ThruPort arterial cannula (Edwards Lifesciences) was placed into the left common femoral artery. The IntraClude endoaortic occlusion balloon (Edwards Lifesciences) was placed in the ascending aorta through the side port. The robotic system was then docked. After cardiopulmonary bypass was initiated, the patient was cooled to 32 °C. Using robotic electrocautery, we were able to dissect away pulmonary adhesions to the pericardium as well as to the right and left atrium. We proceeded with our dissection along the right and left atrium and were able to develop planes between the residual area of pericardium and the epicardium. The IntraClude balloon was then inflated, and the ascending aorta was occluded. A liter of antegrade cardioplegia was then delivered. Intermittent doses of antegrade and retrograde cardioplegia were given every 10 to 15 minutes. Left atriotomy was then performed. We were readily able to identify the LV tumor through the mitral valve. It was well visualized along the posterior aspect (Figure 2A). It did not involve any chordal structures or valvular structures of the mitral valve. The attachment point of the tumor was identified on the trabeculated muscle. A wedge of muscle was transected to remove the mass en bloc with no residual debris or residual remnant of the mass (Figure 2B). After a thorough inspection, we irrigated the left ventricular and left atrial cavity with saline to remove any potential loose debris. We tested the mitral valve, which was competent without regurgitation. The left atrium was closed in a single-layer running suture. The balloon was deflated. After deairing, the LV vent was removed, and the suture line was secured. No residual mass was noted on transesophageal echocardiography. Mitral valve function was normal with no regurgitation. The cross-clamp time and cardiopulmonary bypass time were 58 minutes and 118 minutes, respectively. The patient was extubated and then transferred to the intensive care unit. The postoperative course was uneventful, and she was discharged home on postoperative day 6. The tumor was confirmed as a myxoma by histopathologic examination. On follow-up echocardiography 3 years after the operation there was no evidence of recurrence. A supplemental case video is available (Video).

**Figure 2:**
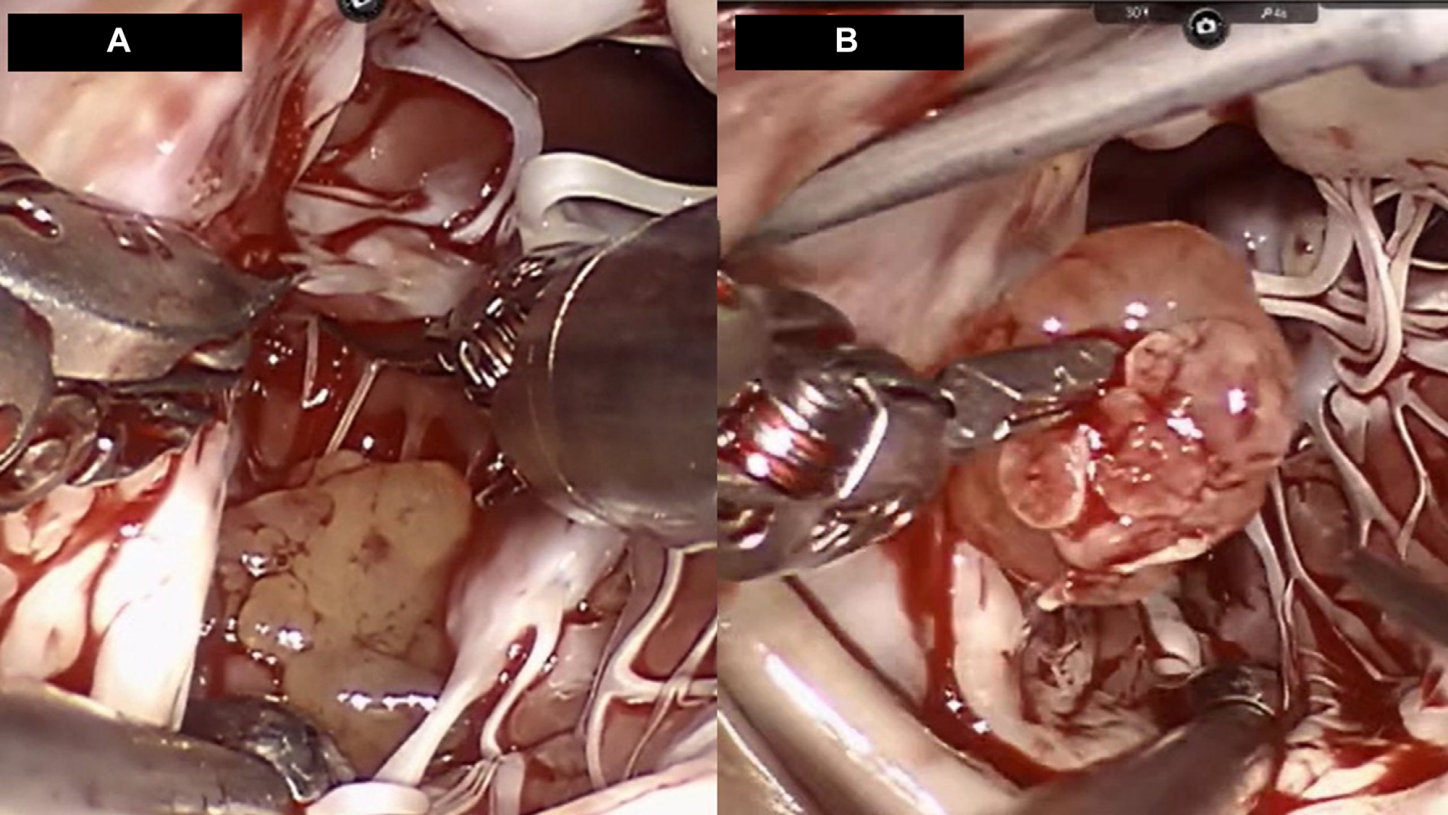
(A) The left ventricular myxoma is well visualized across the mitral valve by the lateral endoscopic approach with robotics technique. (B) The left ventricular myxoma is resected en bloc by fine robotic instruments.

## Comment

Cardiac myxomas are the most common type of benign cardiac tumor, accounting for approximately 50% of all benign cardiac tumors in adults. More than 80% of them are located in the left atrium, and less frequently do they originate in the left ventricle.^2^ Myxomas are typically associated with the risk of embolic phenomena, especially if they are villous or papillary form, which tend to fragment spontaneously. Commonly, a fragmentation travels into the cerebrovascular system and causes stroke, leading to diagnosis of cardiac myxomas. In other cases, they can be discovered incidentally along with various imaging studies. Regardless of the presence of symptoms, surgical removal of myxomas should always be considered, especially in the setting of left-sided lesions, because of the potential risk for systemic embolization.

Unlike atrial myxomas, which are usually easily accessible by atriotomy, LV myxomas are more difficult to expose owing to the location surrounded by complex structures. Traditionally, LV myxoma removal is performed by median sternotomy across the mitral valve, the aortic valve, or the ventriculotomy. Because the risk for damaging surrounding structures or performing incomplete removal can be higher if the exposure is suboptimal, adequate exposure of the myxoma is essential for successful operation.

The first reported case of LV myxoma was in 1959, and since then, more than 70 LV myxoma cases have been reported, and >90% of these cases have been performed through median sternotomy.^3^ Only a few cases have been treated with a minimally invasive approach using thoracoscopic assist.^4^^,^^5^ No other case was found to date that was treated with a robot-assisted approach for a recurrent LV myxoma. Because this patient had a previous operation through right thoracotomy, a sternotomy approach may have been reasonable. However, our most important consideration was optimal visualization to achieve an en bloc resection, given that she had already had recurrence once, which may have been due to incomplete resection or positive margins during her initial operation. Therefore, we concluded that visualization of the LV mass would be best achieved by LEAR. In fact, by using the robotic atrial retractor to “push away” the anterior leaflet as well as the atraumatic ball-tip suction to clear the blood by bedside assistant, we were able to obtain optimal visualization of the LV myxoma between the chordal structures. Based on our experience in this approach, we believe LEAR would be suitable for any LV myxomas, regardless of their location within the left ventricle.

In conclusion, we successfully performed reoperative LV myxoma excision by using our LEAR technique without damaging any surrounding structures. We believe that LEAR provided excellent visualization of the LV myxoma and enhanced a complete en bloc resection, and it was accomplished safely without any perioperative complications.

The Video can be viewed in the online version of this article [https://doi.org/10.1016/j.atssr.2022.10.009] on http://www.annalsthoracicsurgery.org.
